# On Phlegmasia Dolens

**Published:** 1814-09

**Authors:** Francis Moore

**Affiliations:** Ipswich


*For the Medical and Physical Journal.*

### *On Phlegmasia Dolens;* by FRANCIS MOORE, M.D. of Ipswich.

PHLEGMASIA DOLENS, though not a disease of frequent occurrence, is very distressing to the patient, and perplexing to the physician. Until within a few years, it has not been particularly designated and treated of by medical writers. And a great diversity of opinion still subsists among these writers of the proximate cause of the disease.

An interesting case which lately occurred in my own practice, together with an acquaintance with several others which took place in my neighbourhood, have induced me very particularly to consider the symptoms and progress of the disease, with an endeavour to a more satisfactory explanation of its existing and proximate cause, whereby to regulate the method of treatment. I shall relate two cases.

The first was that of a lady of slender and delicate constitution, aged thirty. A few days succeeding parturition, (which was natural, of short duration, and unattended with any peculiar symptoms,) complained of pain in her right side, hip, and back, accompanied with slight rigors and watchfulness. An opiate and antimonial were taken at bedtime, which allayed the pain, and procured sleep.

As the disease progressed she experienced a rigidity and soreness of the abdominal muscles and integuments of that side, attended with a difficulty of moving the lower limb and pains extending to the knee, and at other times to the calf of the leg. The pulse was but little increased in frequency, and but a slight degree of thirst, the tongue perfectly clear. From a knowledge that she had frequently before been affected with rheumatism, I continued the antimonials, with the occasional administration of cathartics and the application of

 fomentations, fomentations, by means of an infusion of bitter herbs inclosed in flannel.

These means proving inefficacious, I had recourse to bark and guaiacum, and kept up a constant irritation on the thigh by blisters, applying another as soon as the first dried. This course evidently increased the local affection. The upper part of the thigh, the inguinal glands, and right iliac region, became more tumefied, which gradually extended to the hypogastrium and labium pudendi.

In the early stages of the swelling, it appeared in ridges and bunches, occasionally assuming a livid, and at other times a purple hue. As it advanced, it appeared more uniformly diffused, exceedingly tender to the touch, hotter than natural, shining, but not much discoloured.

The symptomatic fever which had from the beginning kept pace with the local affection, was now much increased, the pulse small and very frequent, and the thirst considerable. The swelling gradually increased, and the patient, in addition to the other symptoms, sometimes experienced a numbness in the diseased part. With an intention of rousing the action of the absorbents, a volatile stimulating liniment was applied, and in turn hot vinegar; but these had no better effect than blistering.

With the advice of an experienced and highly respectable practitioner, I tried digitalis with a view to its diuretic operation; but, as it failed to produce this effect, and increased the debility, I laid it aside.

During the advanced stages of the disease, the inflammatory appearances became more evident, the pain, heat, soreness, and redness increased, until discharge took place from a ruptured lymphatic in her side, the orifice was about the size of the head of a common pin, situated about an inch from the inferior spinous process of the os ilium.

The fluid discharged on the first day exceeded a pint, resembling a mixture of pus and lymph, its consistence a little thicker than milk, of a pale yellow colour, a little inclined to a faint green, and perfectly inodorous, readily coagulating by admixture with muriate of ammonia, and imperfectly by heat, but not by dilute sulp. acid; specifically heavier than water, and subsiding to the bottom of the vessel, without mixing in any degree; on agitation, uniformly diffusing itself, and producing a milky appearance. The strength of the patient was supported by bark and wine, and the discharge promoted by a wheat-bran poultice.

On the second day the tumour was reduced to one-third of its original size, and the pain and uneasiness had completely subsided. I ordered the vol. liniment to be applied to the remaining remaining hard places, and the continuance of the poultice. Half an hour after, a disagreeable feeling at the stomach and a disposition to fainting ensued: on wiping off the liniment these symptoms passed off.

On the third day a disposition to fainting again occurred, preceded by a disagreeable feeling at the stomach and coldness of the extremities, and accompanied with a creeping and otherwise indescribable sensation along the course of the spine, to near the first or second true rib. To these symptoms succeeded an increased heat and considerable thirst, with a small and frequent pulse.

Suspecting that the sensation experienced along the spine, being in the course of the great lymphatic trunk, might be occasioned either by the absorption of some of the confined matter, or by the sudden determination of the fluids to their natural channel, I examined the side and found the orifice partially closed, which I opened, and by pressure evacuated half a pint of matter resembling that before described. My patient observed she felt relieved at her stomach, and the febrile symptoms immediately disappeared. The matter discharged on the three or four subsequent days contained a large proportion of pus; after which for several days it was perfect lymph.

After about a fortnight the discharge entirely ceased, the tumour disappeared, and the poultice was exchanged for a bandage and compress, and the enfeebled vessels strengthened by astringent lotions and gentle friction, by which means a complete cure has been effected.

The second case occurred in a person of a more robust and healthy constitution, aged twenty-eight, supervening on natural and easy labour. On the second day after parturition she experienced great rigidity of the abdominal muscles and teguments, which gradually became more tense and painful. The surface of the swelling, as in the other case, was unequal, having the appearance of bunches and ridges; the symptomatic fever was considerably violent, which was evinced by a frequent pulse, flushed countenance, and great thirst. These symptoms continued to increase until the end of the fifth week, when suppuration (preceded by strong rigors) commenced from a small opening a little below the navel.

The discharge was very copious, and evidently a mixture of pus and lymph. Ulceration in two or three other parts of the abdomen successively took place. The secretion of milk was almost entirely suppressed, and the child taken from the breast. Suppuration continued about six weeks from its commencement, when the tumefaction had completely subsided; sided; but the patient remained in a very debilitated state for nearly six months.

The disease was in this instance considered by the physician a *consumption*, and from the treatment pursued, it is evident nature had her full operation.

"Mr. White attributes the proximate cause of the disease in question, to an obstruction, detention and accumulation of lymph in consequence of some accident happening during labour, or the continued pressure of the lymphatic vessels by the head of the fœtus."

"Mr. Trye thinks it may be occasioned by pressure, or by the absorption of some acrimonious matter."

Dr. Ferriar thinks it may be produced by some violence during labour, but says "The balance of the circulating fluids is suddenly and violently changed; there are new determinations, new sympathies produced in a state of debility, agitation and anxiety. It cannot therefore surprise us, that, under circumstances so peculiar, a set of vessels commonly exempted from inflammatory affections, should take on an unusual disposition."

Dr. Hull offers the following explanation of its causes. "1st. The increased irritability and disposition to inflammation which prevail during pregnancy, and in a still higher degree for some time after parturition. 2dly. The over distended or relaxed state of the blood-vessels of the inferior part of the trunk, and of the lower extremities. 3dly. Contusions or violent exertions of the muscles about the pelvis and thighs. 4thly. Plethora, occasioned by a suppression or diminution of the lochia, or of the secretion of milk. 5thly. Food taken too freely. And 6thly. Standing or walking too much or too early."

In refutation of Mr. White's opinion, I will state, that in no instance that has come to my knowledge, has the disease preceded parturition.

Mr. Trye's theory is mere supposition. In reply to Dr. Ferriar I will adduce the fact, that phlegmasia dolens as frequently follows natural and easy labour, as difficult and laborious.

Without entering more particularly into an examination of these theories, I think them inadequate to a full and rational explanation of the causes.

After an attentive observation of cases, and a careful examination of the subject, I will humbly offer the following explanation, as the most satisfactory to me.

During gestation the abdominal muscles, their vessels and integuments, are in a state of great preternatural distention; immediately after parturition, when the distending cause is removed, removed, these parts powerfully contract in order to regain their natural dimensions. If this effort be unequally exerted, if it be suddenly excited by the application of cold, if the lymphatic vessels be over distended at the time of plethora, or great debility subsists in the vessels themselves, an interruption and accumulation of the fluid ensues; the great and long-continued accumulation of which, acting as an extraneous and offending cause, will occasion inflammation. In persons of a plethoric and irritable habit, inflammation may quickly supervene; while on the other hand, in a person of a contrary description it may be more tardy in its progress.

This opinion is rendered still farther probable from the consideration of the fact, that inflammation of the mammæ usually occurs during the early stage of lactation. At this period the vessels are incapable of immediately adapting themselves to the sudden accumulation of the fluids, and from their winding direction do not dilate uniformly throughout their whole course, in consequence of which congestion readily occurs. From their unusual and over distended state, inflammation easily takes place on the sudden application of cold.

In those cases which have come to my knowledge, a swelling and enlargement of the parts has uniformly appeared first in the lower part of the abdomen and inguinal glands. That a swelling of the inferior extremities should so frequently succeed to an obstruction of the vessels where they enter the abdomen, will be evident to every one who is acquainted with their course.

In reply to the question, why does not this congestion take place in the sanguineous vessels? it may be answered, that the blood circulates more rapidly, is impelled with greater force, and the vessels do not pass through a congeries of glands.

In the ordinary mode of treatment, much time is lost in the inefficacious use of diuretics; and much mischief and pain produced by the application of blisters and other stimulating remedies. From the low state of sensibility in the lymphatic vessels, inflammation proceeds slowly, and the delay occasioned by the neglect of proper means, or by the use of improper remedies, adhesive inflammation takes place, which often aggravates the disease and renders the cure very tedious and difficult.

From the view here taken of the subject, I am fully disposed to regard it as a local disease, and decidedly recommend the early application of a large emollient poultice, which, by its relaxing and resolving power, will, in a great majority of cases, prevent the formation of a distressing and tedious disease; and, where it does not produce this most desirable effect, I should recommend its continuance with an intention intention of producing early suppuration, which I think, next to resolution, the most speedy and safe termination of the disease.

NOTE. The distinguished physician and philanthropist, Dr. Lettsom, of London, makes some remarks on this disease, in a late letter to one of the editors, which it seems proper to introduce in this place. "The disease, called, by the French writers, 'dépot de lait,' has been elaborately written on by Drs. White, Ferriar, and Hull, of Manchester. The first severe case of it came under my care about thirty-five years ago; but I have since frequently seen it. It is painful and alarming in some instances, though it soon yields to medical treatment. If the pain be considerable it may be allayed by fomentation of poppies; but usually, an embrocation of spiritus mindereri, or weak solution of acetate of lead, with the aid of gentle pressure by a flannel or calico bandage, soon dissipates the swelling; and with the internal use of tonics, such as infusum rosæ, or cinchonæ, the strength and general health are soon restored." *New-Eng. Jour.*

